# Formulation and Evaluation of Self-Nanoemulsifying Drug Delivery System Derived Tablet Containing Sertraline

**DOI:** 10.3390/pharmaceutics14020336

**Published:** 2022-01-31

**Authors:** Anroop B. Nair, Bhavna Singh, Jigar Shah, Shery Jacob, Bandar Aldhubiab, Nagaraja Sreeharsha, Mohamed A. Morsy, Katharigatta N. Venugopala, Mahesh Attimarad, Pottathil Shinu

**Affiliations:** 1 Department of Pharmaceutical Sciences, College of Clinical Pharmacy, King Faisal University, Al-Ahsa 31982, Saudi Arabia; baldhubiab@kfu.edu.sa (B.A.); sharsha@kfu.edu.sa (N.S.); momorsy@kfu.edu.sa (M.A.M.); kvenugopala@kfu.edu.sa (K.N.V.); mattimarad@kfu.edu.sa (M.A.); 2 Department of Pharmaceutics, Institute of Pharmacy, Nirma University, Ahmedabad 382481, India; 13mph103@nirmauni.ac.in; 3 Department of Pharmaceutical Sciences, College of Pharmacy, Gulf Medical University, Ajman P.O. Box 4184, United Arab Emirates; sheryjacob6876@gmail.com; 4 Department of Pharmaceutics, Vidya Siri College of Pharmacy, Off Sarjapura Road, Bangalore 560035, India; 5 Department of Pharmacology, Faculty of Medicine, Minia University, El-Minia 61511, Egypt; 6 Department of Biotechnology and Food Technology, Durban University of Technology, Durban 4000, South Africa; 7 Department of Biomedical Sciences, College of Clinical Pharmacy, King Faisal University, Al-Ahsa 31982, Saudi Arabia; spottathail@kfu.edu.sa

**Keywords:** self-nanoemulsifying tablets, nanoemulsion, full factorial design, sertraline, pharmacokinetics, bioavailability

## Abstract

Being a biopharmaceutics classification system class II drug, the absorption of sertraline from the gut is mainly limited by its poor aqueous solubility. The objective of this investigation was to improve the solubility of sertraline utilizing self-nanoemulsifying drug delivery systems (SNEDDS) and developing it into a tablet dosage form. Ternary phase diagrams were created to identify nanoemulsion regions by fixing oil (glycerol triacetate) and water while varying the surfactant (Tween 80) and co-surfactant (PEG 200) ratio (S_mix_). A three-factor, two-level (2^3^) full factorial design (batches F1–F8) was utilized to check the effect of independent variables on dependent variables. Selected SNEDDS (batch F4) was solidified into powder by solid carrier adsorption method and compressed into tablets. The SNEDDS-loaded tablets were characterized for various pharmaceutical properties, drug release and evaluated in vivo in Wistar rats. A larger isotropic region was noticed with a S_mix_ ratio of 2:1 and the nanoemulsion exhibited good stability. Screening studies’ data established that all three independent factors influence the dependent variables. The prepared tablets displayed optimal pharmaceutical properties within acceptable limits. In vitro sertraline release demonstrated from solid SNEDDS was statistically significant (*p* < 0.0001) as compared to pure sertraline. Differential Scanning Calorimetry and X-Ray Diffraction data established the amorphous state of the drug in SNEDDS formulation, while FTIR spectra indicate the compatibility of excipients and drug. Pharmacokinetic evaluation of the SNEDDS tablet demonstrated significant increment (*p* < 0.0001) in AUC_0-__α_ (~5-folds), C_max_ (~4-folds), and relative bioavailability (386%) as compared to sertraline suspension. The current study concludes that the solid SNEDDS formulation could be a practicable and effective strategy for oral therapy of sertraline.

## 1. Introduction

In the pharmaceutical product development field, there exist a wide variety of drug substances including potential drug candidates which are often limited by poor aqueous solubility, despite their high therapeutic efficacy. Thus, achieving desired drug concentration at the target site is a major challenge typically faced by the formulation and development scientists. Sertraline, a selective serotonin reuptake inhibitor, is currently used as an anti-depressant, belongs to biopharmaceutics classification system (BCS) class II, has poor aqueous solubility (3.5 mg/L), high lipophilicity (log P 5.1), and low oral bioavailability of approximately 44% [1,2]. Administration of drugs through the oral route is most favored over other modes of drug delivery chiefly due to convenience of self-administration, safety, pain avoidance, high patient compliance, low cost, and flexibility in accommodating different types of drugs [3]. Sertraline is gradually absorbed after oral administration however coadministration with food was shown to increase maximum plasma drug concentration (C_max_) by nearly 25% and decrease time to reach peak concentration (T_max_). Moreover, sertraline undergoes extensive hepatic biotransformation via oxidative and glucuronidation pathways [4].

Various approaches like solid dispersion [5], micronization [6], pH modification [7], crystal modification [8], and self-emulsifying drug delivery systems [9] have been evaluated for enhancing the low solubility and dissolution rate of hydrophobic drugs. Modification of physicochemical properties using typical approaches such as salt formation has inherent demerits. Therefore, the probable reason for the low bioavailability of hydrochloride salt might be due to its conversion to a less soluble freebase which further leads to the formation of aggregates in the gastrointestinal tract, thereby hindering the oral absorption of the drug. Few attempts have been carried out to improve the therapeutic efficacy of sertraline by formulating it into solid lipid nanoparticles [10] as well as loading it into inorganic micro and mesoporous materials [11]. Alternatively, the transdermal delivery approach has also been investigated [12,13].

Lipid-based preparations such as self-emulsifying drug delivery system (SEDDS), self-microemulsifying drug delivery system, and self-nanoemulsifying drug delivery system (SNEDDS) have been largely explored in many studies to augment the bioavailability of highly lipophilic drugs [14,15,16,17]. The SNEDDS encompasses isotropic mixtures of oil, surfactants along with one or more hydrophilic co-solvents or co-surfactants [18]. These nanoemulsion preconcentrates or anhydrous nanoemulsion systems instantaneously emulsify, when dispersed in an aqueous medium under mild agitation to form o/w nanoemulsion with globule size < 100 nm [19]. The spontaneous emulsification process eventually reduces particle size, maximizing surface area hence resulting in enhancement of solubility, dissolution, and bioavailability. Furthermore, these lipid formulations in a liquid state can be readily converted into solid-state powder, granules, beads, and pellets by adsorbing on a solid carrier thereby enabling them to develop as unit dosage forms suitable for peroral administration [20]. The major benefits of conversion to solid dosage forms are high dose precision, lowest variability, portability, stable and accurate dosing. Solidification of liquid SNEDDS (L-SNEDDS) provides additional benefits such as good thermodynamic stability thereby preventing supersaturation of drug [21]. On the other hand, the nanoemulsions are thermodynamically unstable colloidal systems susceptible to time-bound physical stability problems similar to coarse emulsions viz., creaming, coalescence, and phase breaking [22]. The stability of nanoemulsions is also influenced by the temperature as well as pH [23]. It was reported that the chemical properties of various additives included in the formulation can potentially influence the stability as well as in vitro release profile of the nanoemulsions [24].

In SNEDDS, suitable excipients such as oil, surfactant, and co-surfactant are chosen based on their ability to form nanoemulsion spontaneously in the gastrointestinal environment after oral administration [25,26]. Moreover, in situ solubilized drugs formed in the lumen of the GIT can be subsequently absorbed via the lymphatic system evading the hepatic first-pass metabolism [27]. Adsorbing the L-SNEDDS in a suitable carrier can potentially convert the encapsulated drug molecule from crystalline to an amorphous state and hence an increase in the aqueous solubility of the drug [28]. In contrast to other studies, the primary objective of the research was to conduct systematic and thorough investigations to examine the practical feasibility of SNEDDS to improve the oral bioavailability of sertraline. The liquid SNEDDS was later modified to solid SNEDDS (S-SNEDDS) and patient-friendly tablet dosage form based on a full-factorial statistical design of experiment technique for future clinical application via oral therapy. This novel methodology has the prospect of potential industrial scale-up due to the generation of flowable, coherent, and compactable powdered form, fewer manufacturing steps, and equipment, generally regarded as safe (GRAS) approved excipients, avoidance of organic solvents, and therefore less production expense. In addition, in vivo evaluation of selected formulation was studied in Wistar rats, and to assess the relative bioavailability, pharmacokinetic parameters were compared with pure sertraline.

## 2. Materials and Methods

### 2.1. Materials

Sertraline hydrochloride, polyvinylpyrrolidone K30, microcrystalline cellulose, croscarmellose sodium (Ac-Di-Sol^®^) were procured from Torrent Pharmaceuticals, Gandhinagar, India. Kollidon^®^ VA 64 and Solutol HS 15 were received from BASF, Ludwigshafen, Germany. The β-cyclodextrin, castor oil, corn seed oil, crospovidone, glycerol monooleate, isopropyl myristate, L-hydroxypropyl cellulose, magnesium stearate, oleic acid, poloxamer 188, polyethylene glycol 200 (PEG 200), polyethylene glycol 400 (PEG 400), propylene glycol, sodium starch glycolate, sorbitan monolaurate (Span 20), sorbitan monoleate (Span 80), sunflower oil, and talc were commercially acquired from Central Drug House, Mumbai, India. Glycerol triacetate was purchased from HiMedia, Mumbai, India. Labrafac^TM^ WL 1349 and Labrasol^®^ were donated by Gattefosse, Saint-Priest Cedex, France. Lemon oil, orange oil, and coconut oil were procured from Astron Chemicals, Ahmedabad, India. Capryol^®^ 90 and Cremophor^®^ EL were donated by Piramal Healthcare, Mumbai, India. Soyabean oil, Tween 40, and Tween 80 were obtained from Chemdyes Corporation, Vadodara, India.

### 2.2. Quantification of Sertraline

Analysis of sertraline from various samples was carried out by minor modification of the previously reported high-performance liquid chromatography (HPLC) method [29]. The system is comprised of the Shimadzu Prominence HPLC (DGU-20A5, Tokyo, Japan) attached with a monolithic C_18_ HPLC column (Zorbax, 150 mm × 4.6 mm). The quantification of the analyte was carried out by connecting to a fluorescence detector maintained at an excitation wavelength of 260 nm and emission at 310 nm. The extraction of sertraline and subsequent analysis was performed using a solvent mixture constituted of acetonitrile and water (80:20% *v*/*v*) maintained at a flow of 1 mL/min. The volume of injection was set at 50 μL, and the retention time was noticed at 12.2 min. Linear regression analysis demonstrates acceptable linearity between the sertraline concentration of 5–500 ng/mL (r^2^ = 0.9992). The method was validated as per ICH Q2 guidelines [30], which demonstrated the limit of quantification (LOQ) and the limit of detection (LOD) as 7.90 ng/mL and 3.60 ng/mL, respectively. The coefficient of variation was estimated between 1.26–4.84% and the recovery of sertraline from plasma was found to be 96.35 ± 1.3%.

### 2.3. Liquid Self-Nanoemulsifying Drug Delivery System (L-SNEDDS)

#### 2.3.1. Preliminary Studies for Components of L-SNEDDS

The solubility of sertraline in various oils (Capryol 90^®^, castor oil, coconut oil, corn seed oil, glycerol triacetate, isopropyl myristate, Labrafac™ WL 1349, lemon oil, oleic acid, olive oil, orange oil, soyabean oil, and sunflower oil), surfactants (Cremophor^®^ EL, Labrasol^®^, solutol HS 15, Span 20, 80, Tween 40 and 80), and co-surfactants (glycerol monooleate, PEG 200, PEG 400, propylene glycol) was estimated by equilibrium solubility method. To experiment, an extra quantity of the drug was placed in vials holding 2 mL of each of the excipients. The vials were shaken using an orbital shaker at 40 rpm at 37 ± 0.2 °C for 72 h. After 72 h, the vials were centrifuged at 4000 rpm for 10 min, filtered, suitably diluted with methanol, and analyzed by HPLC.

#### 2.3.2. Creation of Ternary Phase Diagram

A ternary phase diagram was drawn using the titration method as mentioned elsewhere [31]. The physical characteristics of the nanoemulsion were noted on the individual axis of the phase diagram depicting percentage water, oil, and S_mix_ at fixed weight ratios, respectively. To experiment, different ratios (1:1, 1:2, 1:3, 2:1, 3:1, 4:1) of S_mix_ combinations were prepared. A set of oil/water mixtures were prepared at all possible ratios (9:1, 8:2, 7:3, 6:4, 5:5, 4:6, 3:7, 2:8, 1:9) and titrated with S_mix_ in 5% increment up to 100% to establish the nanoemulsion region. The total volume of S_mix_ consumed was expressed as % *v/v* and phase diagrams were created using Chemix School software (version 3.60, Bergen, Norway) to obtain the nanoemulsion region.

### 2.4. Thermodynamic Stability and Dispersibility Studies of L-SNEDDS Preparations

Thermodynamic stability studies were performed based on centrifugation test (5000 rpm for 30 min), heating-cooling cycle (45 °C and at 0 °C for 48 h), and freeze-thaw cycle (−21 °C and 21 °C for 24 h) [32]. The lack of phase separation indicates the stability of the preparation. Dispersibility test which demonstrates the efficiency of self-emulsification tendency of SNEDDS was evaluated using a USP dissolution type II apparatus (Paddle), where 1 mL of SNEDDS was separately added to distilled water or 0.1 N HCl (500 mL) kept at 37 ± 0.5 °C and paddle rotated at 50 rpm [33]. In vitro behavior of the preparations was visually checked based on the following grading system [34]: (A) Spontaneous formation (<1 min) of nanoemulsion with a transparent or slight bluish look; (B) Spontaneously forming with low transparent microemulsion, with a bluish color; (C) Formation of milky emulsion (<2 min; (D) Dull, greyish white emulsion with somewhat oily appearance (>2 min); and (E), Formulation with low emulsification but had big oil globules appearing on top.

### 2.5. Screening of Formulations by Full Factorial Design

Based on the results of the ternary phase diagram and preliminary studies like screening of components, thermodynamic stability, and dispersibility studies, the independent variables with their effective concentration were identified. From the suitable statistical experimental designs, the full factorial design was selected. The L-SNEDDS were prepared based on the statistical design of the experiment applying a three-factor, two levels (2^3^) full factorial design (FFD) using Design-Expert software (Stat-Ease, Version 12, Minneapolis, USA) by selecting the amount (milligram) of glycerol triacetate (X_1_), Tween 80 (X_2_) and PEG 200 (X_3_) as independent variables, while dissolution efficiency % (Y_1_), globule size in nm (Y_2_) and self-emulsification time (SEF) in secs (Y_3_) as responses and dependent variables (Table 1).

Response surface analyses were performed to find the influence of various independent factors on the observed dependent variables or responses. A set dose of sertraline hydrochloride (50 mg) was added to oil, S_mix_ at room temperature under constant stirring in a vortex mixer to obtain a homogenous mixture. The responses were statistically analyzed utilizing the one-way ANOVA method. The statistical differences between data displaying *p* < 0.05 were selected as significant. The most favorable formulation was chosen by factorial design, which shows maximum % dissolution efficiency, low globule size, and less self-emulsification time. To evaluate the authenticity of the created mathematical model, the validation of the model was evaluated by checkpoint batch. For these formulations, all the three dependent variables (Y_1_–Y_3_) were evaluated as per established mathematical models and experimental techniques.

### 2.6. Characterization of Designed Batches of L-SNEDDS

#### 2.6.1. Determination of Dissolution Efficiency

**Figure 1:**
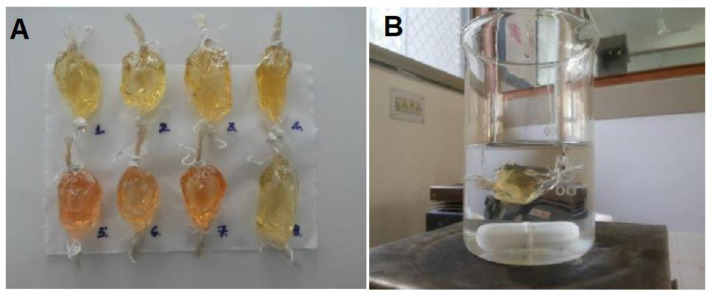
Experimental setup for determination of dissolution efficiency (%); (**A**) SNEDDS formulation filled in dialysis bag; (**B**); dialysis bag in a beaker while experimenting.

A dissolution efficiency test was performed in simulated intestinal fluid (250 mL, pH 6.8) with 10% Tween 80 to achieve sink condition and the dissolution medium was set at 37 ± 0.5 °C and agitated at 100 rpm as presented in Figure 1. The SNEDDS formulation (1 mL) was placed in a previously hydrated dialysis bag (Spectra/Por 4, diameter 25 mm, MW cut-off: 12,000–14,000 Spectrum Inc., Los Angeles, CA, USA). Aliquot volume (1 mL) of the samples was removed at regular time points (5, 10, 15, 30, 45, and 60 min) and an equivalent amount of medium was replaced. The samples taken were diluted suitably, filtered through a 0.2 μm Millex syringe-driven filter unit, and analyzed by HPLC. The dissolution efficiency was calculated according to the equation described below [35].

$$\mathrm{Dissolution}\;\mathrm{efficiency}=\frac{\int_{0}^{t}y\;\mathrm{dt}}{y100\;\left(t₂-t₁\right)}\times 100\%$$

where, the dissolution efficiency is the area under the dissolution curve between time points t_1_ and t_2_ expressed as a percentage of the curve at maximum dissolution; y100, over the same period or the area under the dissolution curve up to a certain time; t, expressed as a percentage of the area of the rectangle described by 100% dissolution in the same time.

#### 2.6.2. Globule Size

To carry out the investigation, 1 mL of L-SNEDDS was placed in a vial and diluted with 20 mL of water. The vial was mildly mixed to generate a fine emulsion and kept for 12 h at room temperature (25 ± 1 °C). The globule size of the nanoemulsion was measured employing a Malvern particle size analyzer (Nano ZS90, Malvern Instruments Ltd., Worcestershire, UK).

#### 2.6.3. Determination of Self-Emulsification Time

To carry out the test, 1 mL from an individual L-SNEDDS preparation was dropped into 500 mL of distilled water taken in a glass beaker and maintained at a temperature of 37 ± 0.5 °C under mild agitation (50 rpm) using a magnetic mixer. The self-emulsification process was visually checked and recorded for the rate of emulsification and subsequent formation of nanoemulsion.

#### 2.6.4. Determination of Viscosity, Zeta Potential, Percentage Transmittance

The viscosity of the undiluted batch (F4) nanoemulsion (0.25 g) was measured using a Brookfield viscometer (LVDVI prime, Middleborough, USA) at room temperature. The zeta potential, as well as polydispersity index, were measured employing a Malvern particle size analyzer (Nano ZS90, Malvern Instruments Ltd., Malvern, Worcestershire, UK). The optical clarity of the emulsion upon dilution was measured as percentage transmittance against double distilled as blank using UV-spectrophotometer (model UV-1800, Shimadzu Corporation, Kyoto, Japan) at 500 nm [36].

### 2.7. Solidification of L-SNEDDS

Solidification of selected L-SNEDDS (Batch F4) was done by the solid carrier adsorption method. Briefly, 20 g of L-SNEDDS formulation was blended with 24 g of microcrystalline cellulose to obtain a wet mixture. Later, 6 g of Aerosil 200 was added to the wet mixture and mixed to obtain S-SNEDDS.

#### Flowability and Compressibility

The flow property of S-SNEDDS was estimated by the conventional static angle of repose (θ) method using Flodex apparatus (Erweka, Heusenstamm, Germany) according to the standard formulae [37]. Similarly, percentage compressibility (Carr’s index) of powder was determined utilizing tap density apparatus (Labindia, Mumbai, India) according to the formulae mentioned in other studies [38]. The flowability of the powder was also interpreted with Hausner’s ratio using the standard equation: Tapped density/bulk density.

### 2.8. Tablet Preparation of S-SNEDDS

For the preparation of S-SNEDDS loaded tablets, listed ingredients (Table 2) were weighed accurately and sifted through sieve number 60. The ingredients were mixed until a uniform mixture was obtained and was again sieved. Lubricant and glidant were added, mixed and the mixture was directly compressed using a 16-station punching machine (Cadmach CMD4, Ahmedabad, India).

### 2.9. Characterization of S-SNEDDS Loaded Tablets

#### 2.9.1. Thickness and Hardness

The thickness, as well as hardness of prepared tablets, were measured by Vernier caliper (1P-67, Mitutoyo, Tokyo, Japan), and tablet hardness tester (125 Series, Erweka Gmbh, Germany), respectively, using three tablets, and an average value was calculated [39].

#### 2.9.2. Friability

The friability of prepared formulations was determined by adding pre-weighed tablets in a Roche friabilator and was allowed to revolve (rotates at 25 ± 1 rpm) for one hundred times according to USP. The same tablets were weighed again after removing dust and calculated the percentage friability [36].

#### 2.9.3. Disintegration

The disintegration time (min) of the tablet was measured employing USP disintegration test apparatus (DT 1000, Labindia, Mumbai, India) in water at 37 ± 2 °C. The time at which the tablet disintegrates completely was noted down and the average time was calculated.

#### 2.9.4. Drug Content

To analyze the drug content, ten tablets were pulverized and the quantity of powder proportionate to 50 mg of sertraline hydrochloride was taken and dispersed in methanol by stirring for 10 min. The solution was filtered using a 0.2 μm filter and assayed by HPLC. The average percentage of drug content was estimated and compared with the total dose.

#### 2.9.5. Drug Release

The percentage release rate of the S-SNEDDS loaded tablet and the pure drug was estimated employing USP dissolution testing apparatus type II (Labindia, Mumbai, India) operated at 75 rpm. The dissolution test was carried out in simulated intestinal fluid (900 mL; pH 6.8) [40] with 10% Tween 80 to maintain sink condition, and the temperature of the dissolution medium was set at 37 ± 0.5 °C using a thermostatically controlled water bath. The selected formulation of sertraline L-SNEDDS, S-SNEDDS, and plain drug equivalent to 50 mg sertraline were used for the comparative evaluation of dissolution data. Aliquot volume (5 mL) of the sample was withdrawn at specified time intervals of 6, 12, 15, 20, 25, 30, 35, 40, 50, and 60 min, and replacements were made with 5 mL of release medium. Each sample was filtered using a 0.2 μm filter and analyzed for sertraline content by HPLC.

#### 2.9.6. Differential Scanning Calorimetry (DSC)

DSC curves of the pure sertraline hydrochloride, microcrystalline cellulose, physical mixture, and S-SNEDDS were obtained by employing a scanning calorimeter furnished with a thermal analysis data system (DSC 60 Shimadzu, Tokyo, Japan). Thermal scanning of the samples placed in hermetically sealed pans was carried out at a temperature ranging between 10 to 300 °C at a heating rate of 20 °C/min using a blank aluminum pan as the reference standard [41].

#### 2.9.7. X-ray Diffraction (XRD)

Diffraction patterns exploring the physical state of the sertraline, blank S-SNEDDS, and drug-loaded S-SNEDDS were studied with the utilization of Bruker AXS D8 Focus P-XRD (Billerica, MA, USA). The drug or formulations were powdered separately and placed in an aluminum holder. The spectral scanning was carried using CU Kα radiation at a similar voltage and current (40 mA) between the range of 2θ angles from 10–40°, with a slow angle scan of 0.01°/min at a sampling interval of 0.02°/s [42].

#### 2.9.8. Fourier Transform Infrared (FTIR)

FTIR spectra of powder samples of pure sertraline and S-SNEDDS were recorded on a Spectrum-GX FTIR spectrophotometer (Perkin Elmer, Waltham, MA, USA). Samples were compressed with potassium bromide (1:10 ratio) at 1 ton/cm^2^ to obtain the disc by employing a hydraulic punching machine [43]. The spectral scanning was performed between 4000–400 cm^−1^.

#### 2.9.9. Scanning Electron Microscopy (SEM)

The morphological characteristics of the powder sample were recorded using an SEM (ESEM EDAX XL-30, Philips, Eindhoven, The Netherlands). Before observation, samples were retained on an aluminum dock using a double-sided adhesive tape which was further coated with gold (~20 nm) to make it electrically conductive in a vacuum. The scanning operation of the SEM was conducted at an acceleration voltage of 15 kV.

### 2.10. Oral Bioavailability Studies

The pharmacokinetic evaluation of sertraline was investigated on male albino Wistar rats (200–250 g) to assess the oral bioavailability differences between the S-SNEDDS and pure drug (control). Animals were caged individually in a well-ventilated room that was maintained at constant temperature (20–24 °C), specified photoperiod (12-h light/12-h dark cycle), and unrestricted access to food and water. Animals fasted for 12 h were classified into two groups (group I and II), each consisting of six rats. Animal experiments were performed following the institutional ethical committee guidelines of animal care (Protocol No. IP/PCEU/FAC/29/2021/40; dated 18/09/2021). A dose equivalent to 5 mg/kg of sertraline or S-SNEDDS was administered as calculated from the standard daily human dose of 50 mg utilizing the equation recommended in the literature [44]. The formulation was prepared as a suspension in 0.5% *w/v* carboxymethyl cellulose and administered to rats orally as a single dose by intragastric gavage in both groups. A blood sample (~200 µL) was drawn at predefined time points (1, 2, 4, 6, 9, 12, and 24 h) post-dosing from retro-orbital plexus of individual rat under anesthesia using isoflurane [45]. Samples were collected in heparin pre-coated tubes and proteins were subsequently precipitated with a similar volume of acetonitrile [46]. It was then centrifuged at 12,000 rpm for 15 min and the supernatant fraction was membrane filtered (0.2 μm). The filtrate (50 μL) was injected into the HPLC system whilst the sample taken at zero time was considered as the baseline value during analysis. The pharmacokinetic parameters of interest included area under the concentration-time curve (AUC_0_–_t_), peak concentration (C_max_), and time to reach peak concentration (T_max_) and were determined by non-compartmental analysis described elsewhere [45].

## 3. Results and Discussion

### 3.1. Preliminary Studies for Components of L-SNEDDS

**Figure 2:**
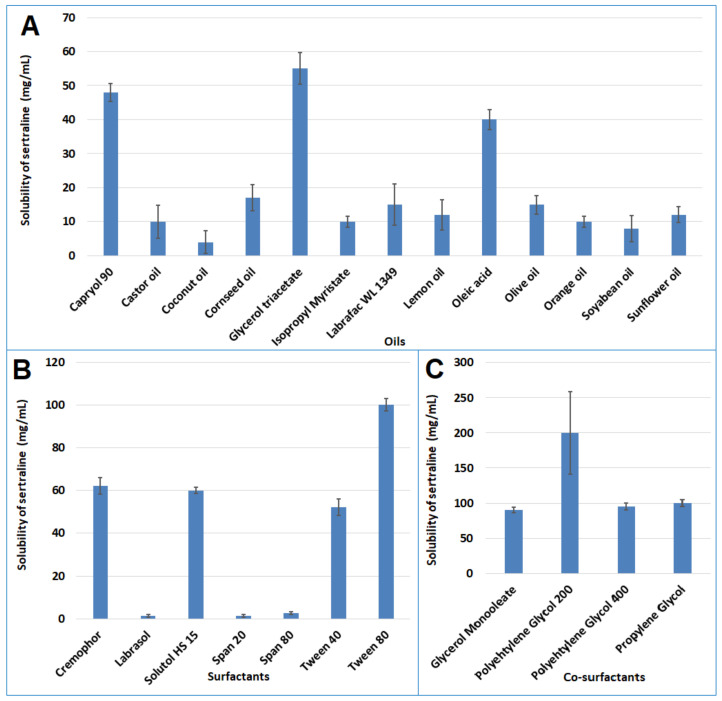
Solubility profile of sertraline in various components of self-nanoemulsifying drug delivery system; (**A**) oils; (**B**); surfactants; (**C**) co-surfactants.

An efficient SNEDDS must spontaneously generate nanoemulsions to enhance the solubility of the drug in the gastrointestinal fluid. Hence the selection of components to formulate SNEDDS is of utmost importance. The study was performed in such a manner to guarantee that the results generated from the prepared SNEDDS closely simulate the in vivo conditions. Indeed, the most widely used excipients were selected for the development of SNEDDS that were not influenced by the alteration in pH and ionic strength, based on the literature [47]. The solubility studies of sertraline were carried out in various oils, surfactants, and co-surfactants was estimated and the solubility profiles are depicted in Figure 2. From the screening study (Figure 2) it was observed that oil (glycerol triacetate; 55 ± 4.62 mg/mL), surfactant (Tween 80; 100.02 ± 2.88 mg/mL) and co-surfactant (PEG 200; 200 ± 58.37 mg/mL) had highest solubility of sertraline. Hence, glycerol triacetate, Tween 80, and PEG 200 were chosen as oil, surfactant, and co-surfactant, respectively.

### 3.2. Construction of Ternary Phase Diagram

**Figure 3:**
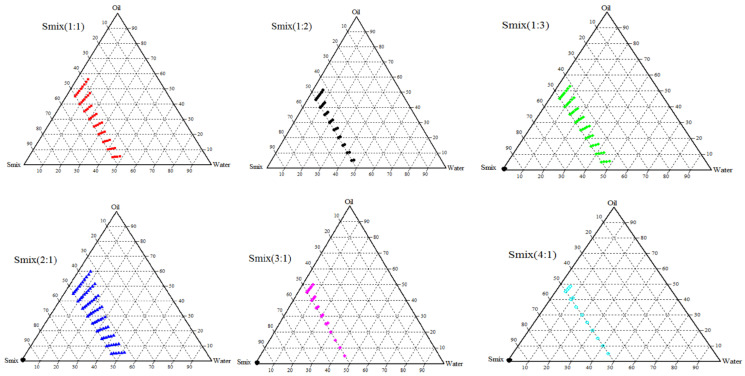
Ternary phase diagrams of glycerol triacetate (oil phase) to identify the nanoemulsion region (as marked by color) with different ratios of S_mix_ (Tween 80: PEG 200).

Nanoemulsion is considered as the isotropic region in the phase diagram wherein transparent and fluid formulations were formed and established through visual observation. The formulation was primarily assessed by evaluating the nanoemulsion regions using pseudo ternary diagrams. It is known that the crucial factor associated with the nanoemulsion formulation is the selection of the type and mass ratio of the surfactant/co-surfactant. Therefore, the oil (glycerol triacetate) and water were fixed and S_mix_ (Tween 80 and PEG 200) ratio was varied (1:1, 1:2, 1:3, 2:1, 3:1, 4:1). The o/w nanoemulsion area in the ternary phase diagram drawn for various S_mix_ ratios is presented in Figure 3.

Nonionic surfactants are advantageous compared to ionic counterparts since they are less toxic and generally have lower critical micelle concentrations. In addition, oral nanoemulsion prepared with nonionic surfactant offers better in vivo stability [48]. The selected nonionic surfactant (Tween 80) has proved to be unaffected due to the change modification of pH and ionic strength besides being typically considered safe. Similarly, the co-surfactant used (PEG 200) has also possessed several advantages as this short-chain alcohol can develop nanoemulsion at minimum surfactant concentration [49]. It can additionally reduce the interfacial tension and enhance the mobility of the interface [50]. The selected surfactant (Tween 80) and co-surfactant (PEG 200) can provide the required HLB value (~10) and therefore can act as an efficient emulsifier in the formulation of nanoemulsion [50].

The size of the nanoemulsion region was considered as the benchmark for the identification and selection of a suitable S_mix_ ratio. It is evident from Figure 3 that the variation of the S_mix_ ratio alters the size of the nanoemulsion region. Initially, when the surfactant quantity was kept constant while increasing the amount of co-surfactant (1:1, 1:2, and 1:3), the nanoemulsion area remained the same (Figure 3). However, the ternary phase diagram showed a larger isotropic region with S_mix_ 2:1. This might be due to the maximum decrease in the interfacial tension of the glycerol triacetate and Tween 80 when the S_mix_ ratio was 2:1, which in turn could have increased the fluidity at the interface to form the nanoemulsion, as reported earlier [32]. Further increase in surfactant concentrations (S_mix_ 3: 1 and S_mix_ 4:1) drastically decreases the nanoemulsion area. A similar observation was also reported wherein the globule size and area of nanoemulsion were modified with alteration in the weight ratio of surfactant/co-surfactant [51]. Based on the comparative evaluation, S_mix_ 2:1 was chosen since it increased the nanoemulsion area.

### 3.3. Thermodynamic Stability and Dispersibility Studies

Diverse techniques such as centrifugation, heating-cooling cycle, and freeze-thaw cycle tests were used to determine thermodynamic stability studies of SNEDDS prepared using S_mix_ ratio 2:1. The results of thermodynamic stability are presented in Table 3. The results here signify that the different ratios of oil, water were thermodynamically stable. Dispersibility test indicates spontaneous formation (<1 min) of the nanoemulsion having a transparent appearance in all ratios of oil/water mixtures prepared using S_mix_ ratio 2:1 (Table 3). As the S_mix_ ratio of 2:1 showed maximum nanoemulsion area, stability, and good dispersibility, this ratio was selected for the application of full factorial design on its nanoemulsion area.

S_mix_, Tween 80 (surfactant) and PEG 200 (co-surfactant); P, passed; A, spontaneous formation (<1 min) of nanoemulsion, having a transparent appearance.

### 3.4. Screening of Formulations by Full Factorial Design

Factorial designs are a statistical design of experiments wherein the influence of multiple factors and their interactions on experimental outcomes are determined. The selected Factorial model (three-factor linear interactions) designs have maximum efficiency in establishing the main effects (X_1_, X_2_, X_3_) and potential interactions (X_1_X_2_, X_2_X_3_, X_1_X_3_, and X_1_X_2_X_3_). Factorial designs have maximum efficiency in establishing the main effects and potential interactions. The main effects indicate the mean outcome of varying one factor at a time from its low to high value. The interaction terms signify how the response alters when multiple factors were entirely transformed. L-SNEDDS were prepared based on the design of the experiment applying a three-factor, two-level (2^3^) full factorial design, and the responses of variables for designed batches (F1–F8) are summarized in Table 4.

#### 3.4.1. Influence of Formulation Factors on Y_1_ (Dissolution Efficiency %)

**Figure 4:**
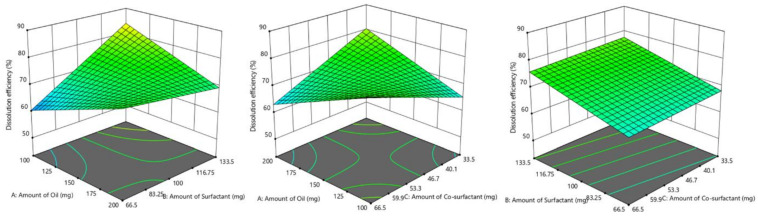
3D surface plot displaying the influence of formulation factors: glycerol triacetate (oil); Tween 80 (surfactant); and PEG 200 (co-surfactant)] on dissolution efficiency % (Y_1_).

The concentration of oil, surfactant, and co-surfactant and related impact on dissolution efficiency are graphically illustrated utilizing design expert software. It is apparent from the 3D surface plot (Figure 4) that the amount of surfactant has a significant effect on dissolution efficiency i.e., with an increase in the amount of surfactant. Dissolution efficiency % also increases whereas the amount of oil and co-surfactant were found to be having a negative effect on Y_1_. From the globule size determination of full factorial batches, it was observed that with a decrease in globule size, dissolution efficiency increases with more surface area.

The fitted polynomial equation to dissolution efficiency % (Y_1_) is:

Y_1_ = 72.15 − 0.198X_1_ + 4.68X_2_ − 0.868X_3_ − 7.06X_1_X_2_ − 7.78X_1_X_3_ + 0.00375X_2_X_3_ − 1.97X_1_X_2_X_3_

The value of X_2_ in the above equation indicates that Tween 80 has a direct influence on dissolution efficiency. As reported extensively in the literature, surfactants are one of the most frequently used excipients in pharmaceutical dosage forms to enhance the solubility of the hydrophobic drugs due to improved wetting which subsequently increases the affinity towards the surrounding solvent [52]. In addition, co-surfactant PEG 200 also in combination with surfactant certainly contributed towards the dissolution rate improvement as indicated by the interaction coefficient, X_2_X_3_.

#### 3.4.2. Influence of Formulation Factors on Y_2_ (Globule Size)

**Figure 5:**
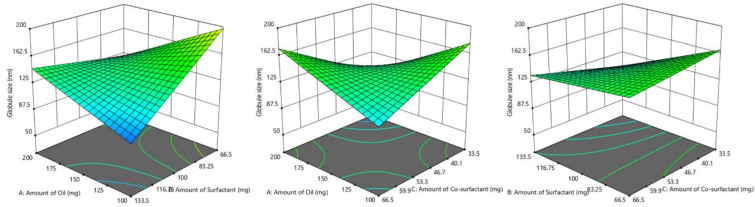
3D surface plot showing the effect of formulation factors: glycerol triacetate (oil); Tween 80 (surfactant); and PEG 200 (co-surfactant)] on globule size (Y_2_).

The relationship between the amount of oil and S_mix_ on globule size is displayed using design expert software. The amount of surfactant showed a significant effect (*p* < 0.05) on globule size i.e., with a decrease in the amount of surfactant, globule size increases. The above statement can also be justified using a 3D surface plot (Figure 5). Similarly, the amount of oil has an opposite effect on globule size, as the amount of oil increases, globule size decreases, while co-surfactant showed a direct effect.

Y_2_ = 139.87 − 5.9X_1_ − 19.79X_2_ + 3.71X_3_ + 32.04X_1_X_2_ + 34.80X_1_X_3_ + 11.88X_2_X_3_ − 0.88X_1_X_2_X_3_

The values X_1_X_2_, X_1_X_3_, X_2_X_3_ demonstrate beneficial or synergistic interactions between oil–surfactant, surfactant–co-surfactant, and oil–co-surfactant to droplet size. It disclosed that the average globule size of the emulsion reduced with increased surfactant concentration due to the creation of a larger oil-water interface [53].

#### 3.4.3. Influence of Formulation Factors on Y_3_ (Self Emulsification Time)

**Figure 6:**
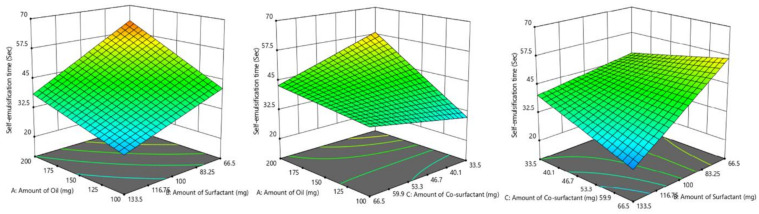
3D surface plot showing the effect of formulation factors: glycerol triacetate (oil); Tween 80 (surfactant); and PEG 200 (co-surfactant)] on self-emulsification time (SEF; Y_3_).

The self-emulsification ability of nanoemulsion is assessed by the time required for emulsification as well as visual observation for transparency. An ideal SNEDDS is expected to disperse spontaneously and completely without any precipitation of the drug under mild stirring in a simulated gastric environment. The effect of the amount of oil, surfactant, and co-surfactant on self-emulsification time is shown by graphical representation. The amount of surfactant showed a significant effect on self-emulsification time i.e., with a decrease in the amount of surfactant, self-emulsification time increases. A similar effect was observed with the co-surfactant PEG 200 as well. While oil has a direct effect on self-emulsification time, an increase in the amount of oil requires more time to emulsify. The above statement can also be justified using a 3D surface plot (Figure 6).

Y_3_ = 42.375 + 8.375X_1_ − 9.875X_2_ − 1.875X_3_ − 2.375X_1_X_2_ − 5.875X_1_X_3_ − 6.125X_2_X_3_ − 0.88X_1_X_2_X_3_

**Figure 7:**
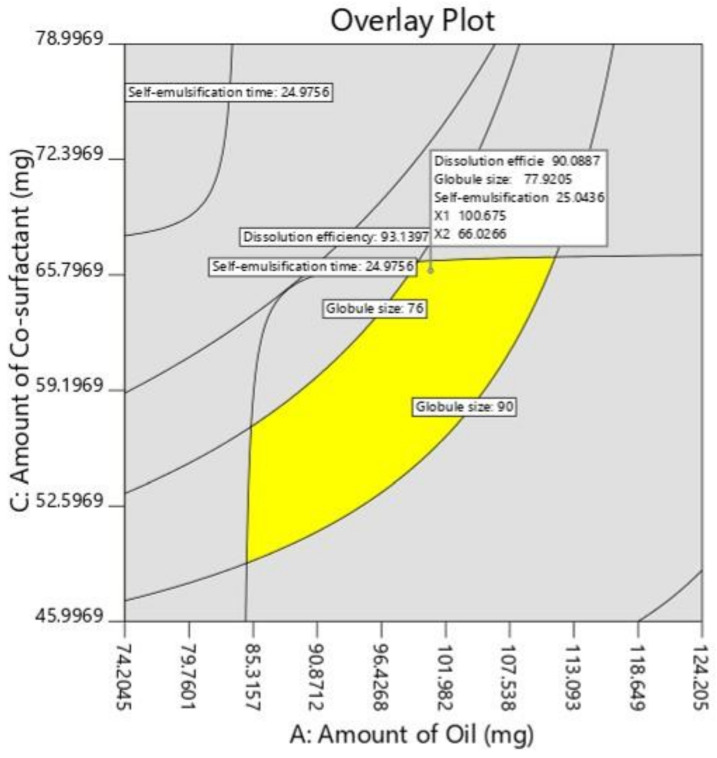
Overlay plot for checkpoint batch using design space.

It was demonstrated that all the prepared batches had self-emulsification times < 1 min and the transparent emulsion was observed, thereby confirming the formation of nanoemulsion. The selected batch (F4) had the lowest self-emulsification time (31.14 ± 2.67 s, Table 4), which might be probably due to the larger surfactant concentration. Surfactant allows rapid transport of water into globules as it reduces the interfacial force that exists between oil and water, causing rupture of emulsifier film and ensuing discharge of enclosed contents into the external fluid. In design expert software, the overlay plot was done and a checkpoint batch was selected (Figure 7) which has a composition near to best batch (batch F4). From Table 5, it was observed that experimental data of the checkpoint batch were nearly close to the predicted values obtained from the design expert software. Hence, it could be concluded that the model selected for identification of the best batch was validated.

#### 3.4.4. Characteristics of Selected Nanoemulsion

Viscosity determination is essential for SNEDDS to monitor and control the physical stability of the system. The selected batch (F4) nanoemulsion showed moderate viscosity (47.7 cPs), which may be probably due to the minimal quantity of oil and S_mix_ used in the developed nanoemulsion. The globule size of the emulsion is a crucial response variable in the self-emulsification process because it decides the biopharmaceutical performance of the developed formulation [54]. It has been disclosed that well-formulated SNEDDS spontaneously formed in simulated gastric conditions under mild stirring. Visual observation of transparent emulsion and transmittance value of 97.25% inferred that formed emulsion is having nanometer globule size range and uniformly dispersed. The Refractive index value (1.42) closer to the refractive index of water (1.33) indicated the isotropic nature of the drug dispersed in the nanoemulsion. The polydispersity index and zeta potential of selected nanoemulsions were 0.422 and −25.5 mV, respectively. The drug content in batch F4 formulation was 0.25 g/1 g of L-SNEDDS.

### 3.5. Solidification of L-SNEDDS

**Figure 8:**
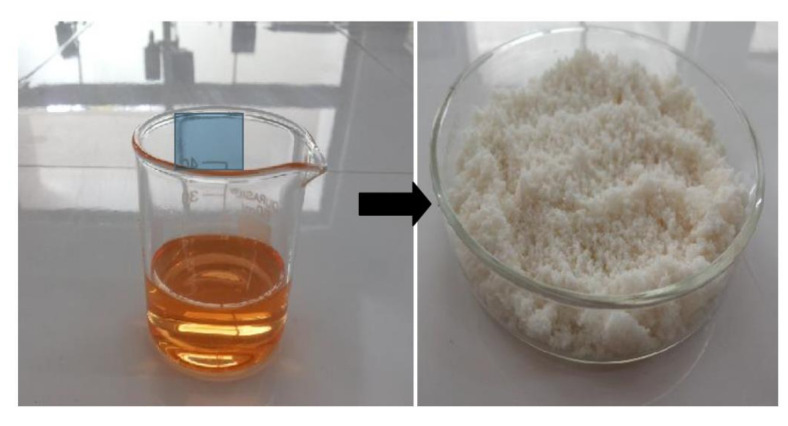
Photographs showing the liquid SNEDDS and solid SNEDDS prepared by solid carrier adsorption method.

S-SNEDDSs have been developed to embed L-SNEDDSs into powders with improved stability and enhanced patient compliance. The formulation approach of converting liquid medication to S-SNEDDS can provide numerous benefits such as good flowability, compactibility, simplified processing steps, improved stability and ease of scale-up, and hence fewer production costs [21]. Solidification of selected L-SNEDDS (batch F4) was done by solid carrier adsorption method using microcrystalline cellulose as a solid carrier. The wet mixture was loaded with Aerosil 200 (as coating material) to get free-flowing powder as depicted in Figure 8. When the L-SNEDDS is added to a highly porous and matted carrier material, it evenly absorbs and adsorbs the drug-loaded liquid dispersion. After the saturation of carrier material, coating material tends to form a particulate film on its surface and eventually absorbs the excess liquid dispersion [55]. Microcrystalline cellulose is considered to be an excellent excipient in the preparation of directly-compressible tablets. Furthermore, microcrystalline cellulose has been widely utilized in drug formulation as a diluent, dispersing agent, emulsion stabilizer, stabilizing agent, and absorbent. Colloidal inorganic solid inert carriers with high surface area such as Aerosil 200 were added to the wet mass in order to extract and redistribute moisture uniformly within the final mixture [56]. Thus, to prepare a dry and free-flowable material, a binary mixture containing sufficient amounts of microcrystalline and Aerosil 200 were taken in a fixed ratio (4:1).

#### Flowability and Compressibility

It is necessary to judge the flow of the materials since sufficient flow is required for uniformity of dosage form. Observed micromeritic properties of prepared S-SNEDDS such as Hausner’s ratio (1.09), and percentage compressibility (9.3) signify that L-SNEDDS ad-sorbed on the surface of the carrier are free-flowing with a high degree of consolidation according to the standard limits [57]. The static angle of repose (Ø) value of 35.6 revealed that liquid-solid powder mixture exhibits acceptable flow properties, which can be further improved by incorporating optimum concentration of glidant such as Aerosil^®^.

### 3.6. Characterization of S-SNEDDS Loaded Tablets

#### 3.6.1. Thickness and Hardness

The tablet thickness will be constant, provided the tablet granulation or powder blend is adequately uniform in particle size and size distribution. The average thickness of the prepared tablet was estimated as 5 mm with a weight of 600 mg. All tablets need a specific amount of strength, or hardness and resistance to friability, to withstand mechanical shocks during manufacture, packaging, and shipping. The average hardness of the prepared tablet was 3.2 kg/cm^2^ and the percentage friability estimated was 0.957, and these values are within the acceptable limits.

#### 3.6.2. Friability

The friability of tablets should always be less than 1%. The results of friability (%) of prepared tablets were found to be 0.91, which is generally considered acceptable for conventional compressed tablets according to the USP.

#### 3.6.3. Disintegration

This test is carried out to measure the time required to disintegrate within the specified time limit when placed in a suitable liquid medium under suitable experimental conditions. The disintegration time of the prepared tablet was 1 min at 37 ± 1 °C.

#### 3.6.4. Drug Content

To evaluate a tablet’s potential for efficacy, the drug content needs to be monitored from tablet to tablet and batch to batch. The drug content of the selected tablet was estimated to be 95.06%, which is within the pharmacopeial acceptance criteria (95 to 105% of the label strength).

#### 3.6.5. Drug Release

**Figure 9:**
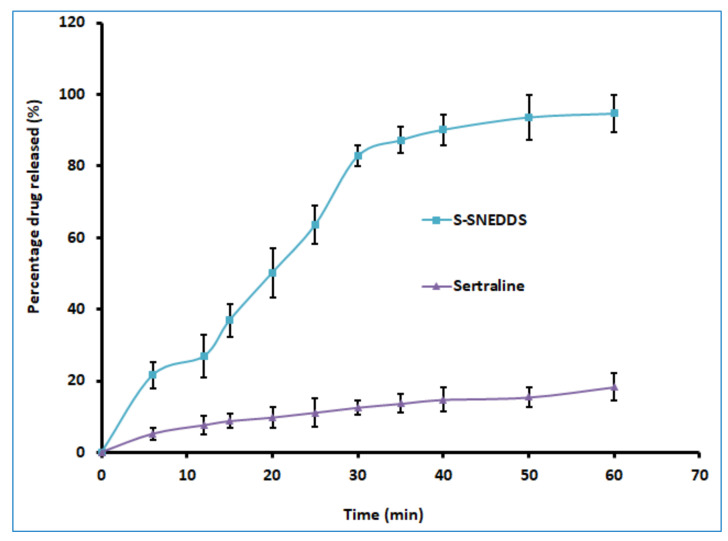
Comparison of release profile of prepared solid self-nanoemulsifying drug delivery system (S-SNEDDS) and sertraline (control). Data represented are mean ± SD (*n* = 6).

Assessment of drug release from formulations is essential as it indicates the possible gastric dissolution and could be a probable tool to forecast the absorption of low aqueous soluble drugs [27]. Therefore, the in vitro release test was conducted for the tablet loaded with SNEDDS and compared with the plain drug having an equivalent amount (50 mg) of sertraline. Performing in vitro dissolution studies in suitable bio-relevant media can provide a more exact simulation of in vivo profile to optimize the dose, formulations, and also to predict bioavailability [58]. Hence, drug release studies were performed in simulated intestinal fluid, which is more suitable for BCS Class II drugs [59]. Two distinct release profiles were exhibited by the tablet containing SNEDDS and pure drug (Figure 9). The amount of drug released in 30 min from profiles of tablets containing SNEDDS and plain sertraline were 82.76% and 12.56%, respectively (Figure 9). Faster drug release profiles (Q>80% in 30 min) confirmed that the SNEDDS-loaded compact tablets met the acceptance criteria as specified in the USP dissolution specification guidance for immediate release dosage forms [60]. Microcrystalline cellulose used in the formulation of S-SNEDDS would have acted as a dispersing agent which facilitates the dissolution rate by rapidly exposing the drug-loaded nanoemulsion droplets to dissolution medium. It is a fact that an increase in the surface area offered by the nanoparticles increases the dissolution rate of poorly water-soluble drugs [61]. However, the release rate was moderately low after 30 min. This could be probably due to the slow release of entrapped L-SNEDDS formulation in the long and narrow pores of Aerosil^®^200 carrier as described elsewhere [62]. Moreover, the drug release from the SNEDDS-loaded compact tablet was complete within 60 min, while it was ~20% with pure drug. The result here indicates that the prepared formulation is capable of increasing the sertraline release and is likely to increase the rate and extent of absorption of this poorly water-soluble drug.

#### 3.6.6. DSC

**Figure 10:**
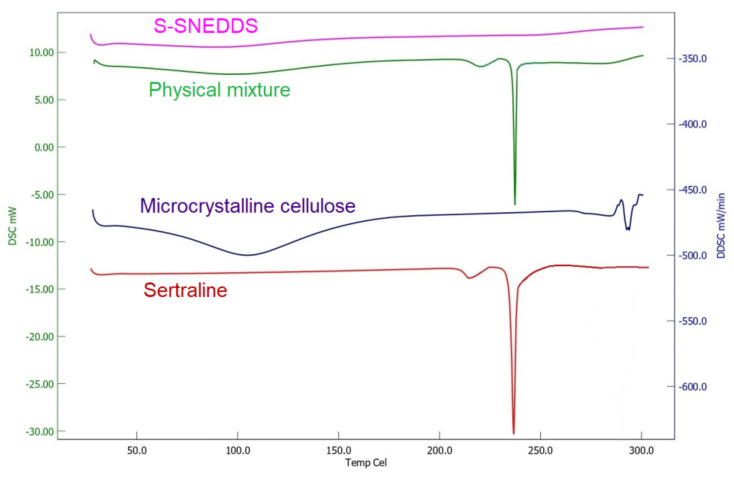
Differential scanning calorimetric thermograms of sertraline, microcrystalline cellulose, physical mixture, and prepared solid self-nanoemulsifying drug delivery system (S-SNEDDS).

Thermo analysis technique utilizing DSC was done to confirm the physical characteristics of the drug resulting from varying orders of molecular transformation from crystalline and amorphous components existing within the powder mixture. The prominent melting endotherm peak showing the crystalline characteristic of sertraline was observed at 244.21 °C (Figure 10). Similarly, the physical mixture also showed a prominent drug peak at the same temperature. However, no sharp peak of the drug was observed in S-SNEDDS which can be interpreted as a change of crystalline to amorphous state which may eventually lead to an improvement of aqueous solubility. The absence of any additional peaks in S-SNEDDS confirms the compatibility between excipients and sertraline in the current formulation.

#### 3.6.7. XRD

**Figure 11:**
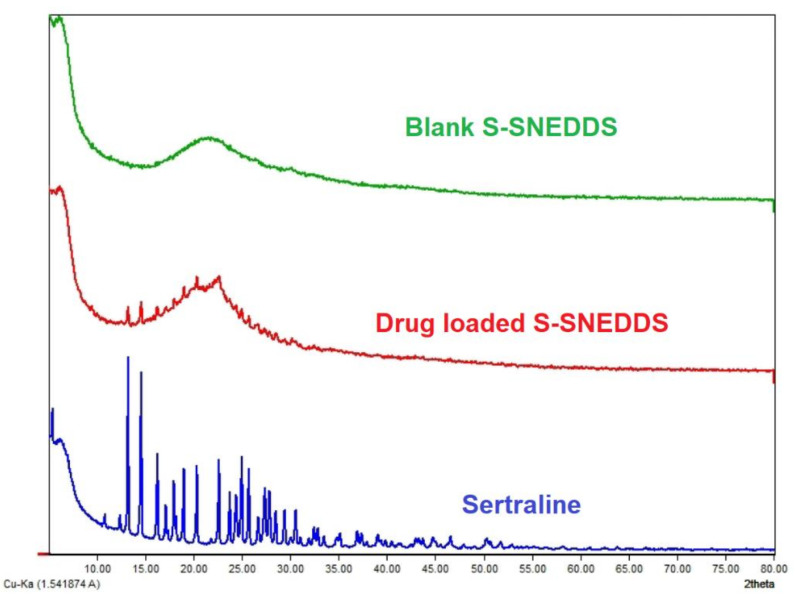
X-ray diffractogram of pure sertraline, prepared blank solid self-nanoemulsifying drug delivery system (S-SNEDDS) and drug-loaded S-SNEDDS.

The XRD pattern of sertraline HCl and drug-loaded-S-SNEDDS is presented in Figure 11. The diffractogram of sertraline showed several characteristic peaks with few sharp and predominant peaks indicating the crystalline state of the drug. However, drug-loaded S-SNEDDS showed a comparatively broad, short, and blunt peak. This might be due to the existence of the drug in an amorphous or molecularly dispersed insolubilized state. The inclusion of additional excipients in the S-SNEDDS resulted in a few extra peaks, which are also present in the blank S-SNEDDS.

#### 3.6.8. FTIR

**Figure 12:**
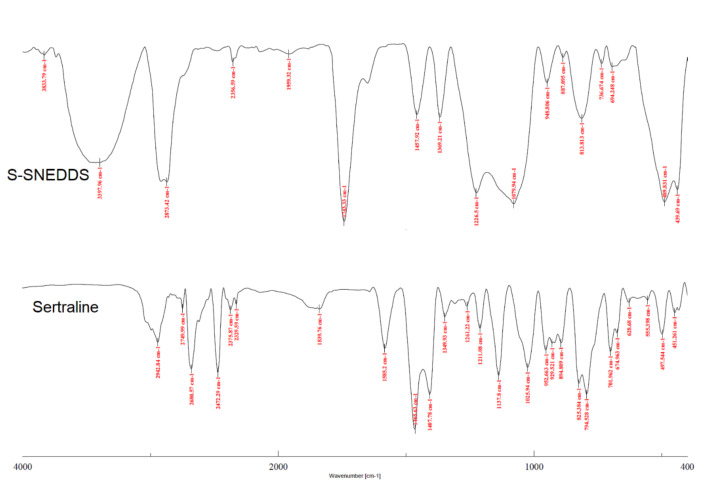
Fourier transform infrared spectroscopy of sertraline and prepared solid self-nanoemulsifying drug delivery system (S-SNEDDS).

The drug excipient interaction in formulations can be assessed using techniques such as FTIR, wherein the drug spectra are compared with the formulation. Any variations such as non-appearance or reduction in spectra could be related to the molecular interaction between tested compounds [63]. The FTIR spectra of sertraline and S-SNEDDS are presented in Figure 12. It is evident from Figure 12 that the FTIR of sertraline showed many dominant peaks like the peaks for C-H stretching (2942.84 cm^−1^), –NH_2_^+^ symmetric stretching (2749.99 cm^−1^), N-H bending vibration (1585.2 cm^−1^), C-H bending vibrations of cyclohexane, CH_3_/CH_2_, asymmetrical scissoring (1465.63 cm^−1^), C-N stretching (1349.93 cm^−1^), CH_3_ twisting (1211.08 cm^−1^), CH twisting (952.663 cm^−1^), CH stretching (894.809 cm^−1^). The FTIR of the SNEDDS formulation showed all characteristic peaks of the drug with either a reduced in intensity, or shift, or change in the pattern of the spectra. The reduced intensities in those peaks might be due to the low concentration of drug compared to the excipients. The additional peaks were observed in the FTIR of SNEDDS formulation with a broad peak at 3397.96 cm^−1^ (hydrogen-bonded OH of microcrystalline cellulose) and 1743.35 cm^−1^ due to other excipients present in the formulation. The strong peak of sertraline N-H bending at 1585.2 cm^−1^ has been observed as a shoulder with a peak of 1743.35 cm^−1^_._ The slight shift in some of the peaks is due to the overlapping of excipients’ peaks. The results suggest the absence of compatibility issues between sertraline and other ingredients used in the composition of prepared S-SNEDDS.

#### 3.6.9. SEM

**Figure 13:**
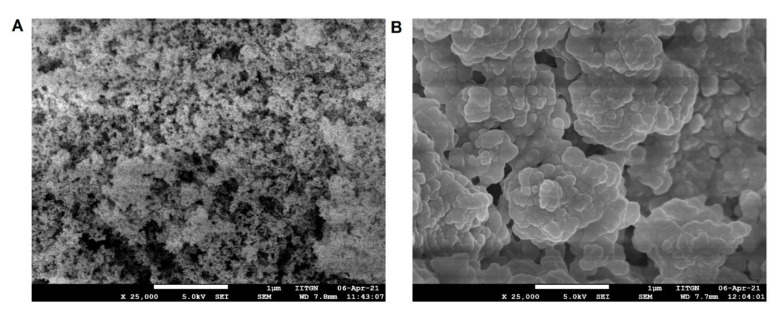
Representative scanning electron microscopy photographs of (**A**) Aerosil 200; and (**B**) prepared solid self-nanoemulsifying drug delivery system (S-SNEDDS). Scale bar represents 1 µm.

The surface features of plain Aerosil 200 (coating material) and selected S-SNEDDS formulation (batch F4) were interpreted utilizing an SEM as illustrated in Figure 13. Aerosil 200 seems to be a highly porous material that exists as loose aggregates composed of colloidal silicon dioxide particles [33]. The SEM picture of S-SNEDDS suggests a change in morphology and becomes spherical particles, but twisted, fused, and marginally uneven in appearance. Isolated minor cracks, dents, and pores are also visible on the matrix structure, which could further enhance the rapid permeation of water and thus allow for rapid dispersibility in the gastrointestinal environment. Further, figures reveal the total adsorption of L-SNEDDS into the carrier materials which were observed noticed by the absence of any oil globules in the S-SNEDDS.

### 3.7. Oral Bioavailability Studies

**Figure 14:**
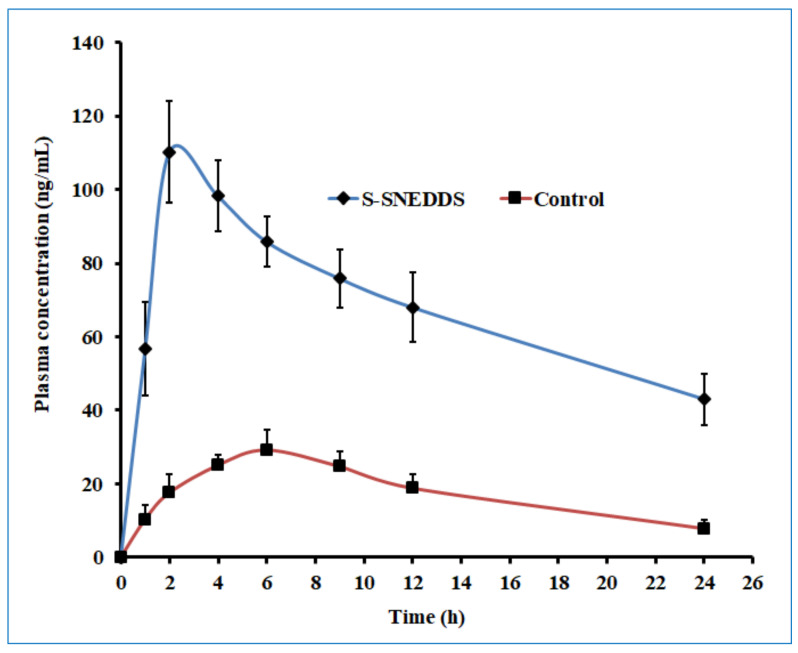
Mean plasma concentration-time profile of prepared solid self-nanoemulsifying drug delivery system (S-SNEDDS) and sertraline (control) in Wistar rats following oral administration. Data represented are mean ± SD (*n* = 6).

The plasma concentrations’ time profile is displayed in Figure 14, and the pharmacokinetic data are shown in Table 6. Evaluation of pharmacokinetic parameters revealed that administration of sertraline as aqueous suspensions resulted in the least plasma concentration–time profile, lowest C_max_ (29.19 ± 5.63 ng/mL), and delayed T_max_ (6 h) compared to S-SNEDDS-loaded tablets (Figure 14). Nevertheless, the calculated AUC_0-__α_ (ng. h/mL) in S-SNEDDS was ~5-fold larger (*p* < 0.0001) than the AUC_0-__α_ obtained for the aqueous suspension of sertraline. The S-SNEDDS-loaded tablet demonstrated a mean value of C_max_ 10.26 ± 13.93 ng/mL, which was ~4-times higher (*p* < 0.0001) than the C_max_ recorded with the similar dose of sertraline given as aqueous suspension. The T_max_ values shown by S-SNEDDS (2 h) were less than T_max_ recorded from the aqueous suspension of sertraline (Table 6). The selected preparations showed enhanced relative bioavailability (386%) as compared to sertraline suspension.

The possible reasons for the observed enhancement in relative bioavailability of sertraline from prepared S-SNEDDS formulation could be many. For instance, increased absorption of sertraline from S-SNEDDS was possible because of enhanced solubilization provided by the surfactant mixture along with the amorphous state of the drug. It has been disclosed that the irregular molecular orientation that exists in the amorphous state causes an improvement of aqueous solubility and therefore enhanced dissolution rate in contrast to their crystalline analog [64]. Different degrees of molecular order can occur simultaneously in S-SNEDDS, leading to the existence of both crystalline and amorphous systems [64]. However, the characteristics of the greater amorphous component present in S-SNEDDS could be advantageous and hence enhance the biopharmaceutical performance of pharmaceutical products [37]. Sertraline S-SNEDDS could form nanoemulsion droplets spontaneously after dispersion in the gastrointestinal tract and have the capacity to absorb quickly, being a BCS class II compound. Moreover, the nano-sized emulsion globules have the potential ability to transport drugs through the transcellular route for gastrointestinal absorption.

Similarly, non-ionic surfactant (Tween 80) might have enhanced the solubility of sertraline through micellar solubilization besides decreasing the interfacial surface tension and thereby enhancing the permeation of the drug via the epithelial barrier. In addition, the lipidic constituents included in the formulation can solubilize a considerable amount of this lipophilic molecule and promote self-emulsification, besides having the capacity to promote a portion of drug transferred via the intestinal lymphatic system, thus promoting gastrointestinal absorption. Overall, it can be concluded from the in vivo pharmacokinetic evaluation that prepared S-SNEDDS formulation significantly improved solubility, dissolution, and absorption, which in turn resulted in greater bioavailability of sertraline.

## 4. Conclusions

To improve the solubility of the poorly water-soluble drug, sertraline, the S-SNEDDS approach was employed. Initially, screening of nanoemulsion formulation was carried out by examining the influence of the quantity of oil, surfactant, and co-surfactant on dissolution efficiency, globule size, and self-emulsification time. The selected liquid SNEDDS formulation (batch F4) was converted to a flowable powder using microcrystalline cellulose as a solid carrier and Aerosil 200 as coating material. The powder was compressed into tablet dosage to integrate the bioavailability enhancement of sertraline besides solving the formulation challenges typically associated with liquid lipid formulations. A rapid and complete drug release noticed in S-SNEDDS formulation met the acceptance criteria specified in the USP dissolution specification guidance for immediate release dosage forms. DSC and XRD data signify the complete transformation of crystalline sertraline in the prepared formulation, which might be contributing towards the improvement of solubility and thereby dissolution enhancement of the drug. The FTIR study confirms no interaction between the drug and excipients used. In vivo data demonstrate a significant (*p* < 0.0001) difference in the C_max_, and AUC_0-__α_ by prepared S-SNEDDS as compared to control, indicating improvement in oral absorption of sertraline. In conclusion, the results obtained here demonstrated the feasibility of S-SNEDDS to enhance the oral bioavailability and thereby the clinical efficacy of sertraline.

## Figures and Tables

**Table 1:** 2^3^ Full factorial design indicating both independent and dependent variables.

Batch No.	Amount of Oil (mg) (X_1)_		Amount of Surfactant (mg) (X_2)_		Amount of Co-Surfactant (mg) (X_3_)	
	Coded Values	Actual Values	Coded Values	Actual Values	Coded Values	Actual Values
F1	−1	100	−1	66.5	−1	33.5
F2	−1	100	−1	66.5	+1	66.5
F3	−1	100	+1	133.5	−1	33.5
F4	−1	100	+1	133.5	+1	66.5
F5	+1	200	−1	66.5	−1	33.5
F6	+1	200	−1	66.5	+1	66.5
F7	+1	200	+1	133.5	−1	33.5
F8	+1	200	+1	133.5	+1	66.5

Responses: Y_1_ = % Dissolution efficiency; Y_2_ = Globule size (nm).

**Table 2:** Composition of the tablet preparation of solid self-nanoemulsifying drug delivery system.

Ingredients	Category	Quantity (% *w*/*w*)	Amount in (mg)
The powder contains 50 mg of the drug	Drug loaded solid self-nanoemulsifying drug delivery system	83.33	500
l-hydroxypropyl cellulose	Binder	8.67	50
Croscarmellose sodium	Disintegrant	6	36
Magnesium stearate	Lubricant	1	6
Talc	Glidant	1	6

**Table 3:** Results of the thermodynamic study and dispersibility test of various liquid self-nanoemulsifying drug delivery systems prepared using S_mix_ ratio of 2:1.

S_mix_ Ratio	Oil: Water Ratio	% *w*/*w*			Thermodynamic Stability			Dispersibility Test	
		Oil	Water	S_mix_	Heating Cooling Cycles	Centrifugation	Freeze-Thaw Cycles	Water	0.1 N HCl
2:1	9:1	58.05	6.45	35.5	P	P	P	A	A
		56.25	6.25	37.5	P	P	P	A	A
	8:2	48.48	12.12	39.4	P	P	P	A	A
		47.04	11.76	41.2	P	P	P	A	A
	7:3	41.16	17.65	41.19	P	P	P	A	A
		39.99	17.14	42.87	P	P	P	A	A
	6:4	35.29	23.53	41.18	P	P	P	A	A
		34.29	22.86	42.85	P	P	P	A	A
	5:5	22.73	22.73	54.54	P	P	P	A	A
		20.83	20.83	58.34	P	P	P	A	A
	4:6	16.67	25.00	58.33	P	P	P	A	A
		15.38	23.07	61.55	P	P	P	A	A
	3:7	13.04	30.43	56.53	P	P	P	A	A
		11.54	26.92	61.54	P	P	P	A	A
	2:8	8.33	33.32	58.35	P	P	P	A	A
		7.69	30.76	61.55	P	P	P	A	A
	1:9	4.35	39.15	56.5	P	P	P	A	A
		3.85	34.65	61.5	P	P	P	A	A

**Table 4:** Responses of designed batches for dependent variables (dissolution efficiency %; globule size; and self-emulsification time).

Batch No.	Y_1_ (break)Dissolution Efficiency (%)	Y_2_ (break)Globule Size (nm)	Y_3_Self-Emulsification Time (s)
F1	55.67 ± 2.02	241.51 ± 40.92	38.52 ± 3.11
F2	65.55 ± 1.82	153.8 ± 42. 55	45.05 ± 4.35
F3	75.20 ± 2.18	112.3 ± 35.58	22.78 ± 3.42
F4	92.98 ± 2.92	75.66 ± 24.47	31.14 ± 2.67
F5	81.02 ±2.07	94.15 ± 29.51	58.37 ± 5.14
F6	67.65 ± 2.15	149.2 ± 38.18	68.33 ± 5.48
F7	80.19 ± 3.11	96.65 ± 24.72	59.18 ± 5.22
F8	58.95 ± 2.19	195.7 ± 39.77	18.28 ± 2.46

**Table 5:** Predicted and experimental values of Y_1_, Y_2,_ and Y_3_ variables of checkpoint batch.

Dependent Variables	Experimental Value	Predicted Value
Y_1_ Dissolution efficiency (%)	92.12	90.08
Y_2_ Globule size (nm)	76.03	77.92
Y_3_ Self-emulsification time (s)	29	25.04

**Table 6:** Mean pharmacokinetic parameters of self-nanoemulsifying drug delivery system loaded tablet and plain sertraline (control) in plasma following oral administration in Wistar rats (*n* = 6).

Parameter	SNEDDS	Control
T_max_^a^ (h)	2	6
C_max_^b^ (ng/mL)	110.26 ± 13.93 *	29.19 ± 5.63
AUC_0-__α_ ^c^ (ng.h/mL)	3115.73 ± 482.58 *	685.73 ± 150.64

* Significant difference (*p* < 0.0001) detected in sertraline plasma level in SNEDDS group compared to control. ^a^ time to reach maximum plasma concentration; ^b^ maximum plasma drug concentration; ^c^ area under the plasma drug concentration-time profile curve.

## Data Availability

The data presented in this study are contained within the article.
